# Estimating recurrences prevented and costs avoided with atezolizumab in early non‐small cell lung cancer in the United States

**DOI:** 10.1002/cam4.5462

**Published:** 2022-11-24

**Authors:** Rishika Sharma, Sarika Ogale, Nathaniel J. Smith, Janet Shin Lee

**Affiliations:** ^1^ Maple Health Group, LLC New York New York USA; ^2^ Genentech Inc. South San Francisco California USA

**Keywords:** atezolizumab, cost simulation, early NSCLC, prevention, recurrence

## Abstract

**Background:**

Recurrence of early‐stage non‐small cell lung cancer (eNSCLC) is associated with significant mortality and costs. Atezolizumab (ATZ) was recently approved as adjuvant treatment following resection and platinum‐based chemotherapy for adults with stage II‐IIIA NSCLC with PD‐L1 expression ≥1% after demonstrating significant improvement in disease‐free survival (DFS) relative to best supportive care (BSC) in the IMpower010 trial (NCT02486718). This study evaluated the population‐level impact of ATZ as adjuvant treatment for eNSCLC in the United States by estimating the number and costs of recurrences avoided.

**Methods:**

A Monte Carlo simulation model estimated the cumulative number of recurrences and deaths prevented, along with direct, indirect, and terminal care costs, by treating eNSCLC patients with ATZ compared to BSC. The model included eligible patients treated in any given year and followed over a 5‐year period. Recurrence and mortality rates and costs were based on the IMpower010 data and supplemented by estimates from published literature.

**Results:**

An estimated 4400 eNSCLC patients in the United States were eligible for adjuvant ATZ in any given year, of whom 2387 would experience recurrence within 5 years with BSC. Following the introduction of ATZ, 1030 (95% confidence interval [CI]: 1023, 1036) recurrences and 369 (95% CI: 362, 376) deaths would be avoided with estimated reductions in cumulative recurrence‐related direct, indirect, and terminal care costs of $785 million, $15 million, and $32 million, respectively, over a 5‐year time horizon.

**Conclusions:**

Adjuvant ATZ is estimated to prevent a significant number of recurrences and reduce the economic burden of eNSCLC.

## Graphical abstract

Adjuvant atezolizumab is estimated to prevent a significant number of recurrences and reduce the economic burden of early non‐small cell lung cancer.

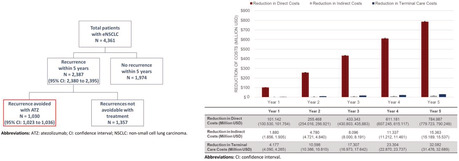

## 1 INTRODUCTION

Approximately 50% of patients diagnosed with non‐small cell lung cancer (NSCLC) have early‐stage NSCLC (eNSCLC), including localized (stages I and II) or locally advanced (stage III) disease.^1^ Surgery is the treatment of choice for eNSCLC and, until recently, treatment guidelines have recommended curative surgery with adjuvant platinum‐based chemotherapy. However, recurrences post‐resection are common, with over 45% of patients experiencing a recurrence within 5 years.^2^, ^3^, ^4^, ^5^, ^6^, ^7^ Further, recurrences are associated with significant morbidity and mortality: the 5‐year overall survival for patients with a recurrence after surgical resection and adjuvant chemotherapy is 35.6%.^2^ Distant recurrences affecting the central nervous system (CNS), in particular, are associated with a high clinical burden, increased risk of death, and impairments in health‐related quality of life.^8^

Recurrences among eNSCLC patients are also associated with a substantial economic burden, resulting in significant health care resource utilization.^2^ In addition, real‐world utilization of adjuvant chemotherapy ranges from 27% to 50% among patients with resected stage II‐III NSCLC,^9^, ^10^ suggesting a significant unmet treatment need among these patients. Highly effective and well‐tolerated treatments in the peri‐operative setting are needed to prolong disease‐free survival (DFS) and overall survival (OS).

The phase 3 clinical trial IMPower010 (NCT02486718), atezolizumab (ATZ; Tecentriq®, Genentech Inc., South San Francisco, CA, USA), a PD‐L1 inhibitor, demonstrated significant reductions versus best supportive care (BSC) in the rate of recurrence among patients with completely resected stage IB to IIIA eNSCLC following adjuvant platinum‐based chemotherapy.^11^ Stratified hazard ratios (HRs) for DFS were 0.66 (95% confidence interval [CI] 0.50–0.88; *p* = 0.0039) in patients with stage II‐IIIA eNSCLC with PD‐L1 expression of ≥1%, 0.79 (95% CI 0.64–0.96; *p* = 0.020) for all patients with stage II–IIIA disease, and 0.81 (95% CI 0.67–0.99; *p* = 0.040) for patients with stage IB‐IIIA disease.^11^ Consequently, ATZ was recently approved by the Food and Drug Administration (FDA) for use as adjuvant treatment following resection and platinum‐based chemotherapy for adult patients with stage II to IIIA NSCLC whose tumors have PD‐L1 expression on ≥1% of tumor cells.^12^

Given the high unmet need in this patient population, evaluating the potential clinical and economic benefits provided by ATZ to both eNSCLC patients and overall society would help payers and other key stakeholders make population‐level decisions while facing budget constraints. Until real‐world data are available to evaluate long‐term treatment outcomes, simulation modeling can provide insight on the potential future benefits of treatment and has previously been used in other cancers.^13^ The objective of this study was to estimate the population‐level health and economic benefits associated with ATZ as adjuvant treatment for patients with eNSCLC in the United States.

## 2 MATERIALS AND METHODS

### 2.1 Overview

A Monte Carlo simulation model was used to estimate the cumulative number of recurrences and deaths prevented by treating eligible patients with eNSCLC with ATZ compared with BSC in the United States. The model was based on a previously published study estimating the cumulative number of recurrences and deaths avoided by treating eligible patients with HER+ stage I to III breast cancer with adjuvant trastuzumab.^13^ Monte Carlo models sample probabilistically from input value distributions over a set number of simulations to predict numerical outcomes of an uncertain event. The model was static (representing a particular point of time) and included a cohort of patients treated for 1 year, followed over a 5‐year time horizon. The base case model included adult patients (aged ≥20 years) with resected stage II to IIIA NSCLC with PD‐L1 expression of ≥1% who received adjuvant chemotherapy, consistent with the FDA label.^12^ The economic costs associated with the recurrence and mortality events avoided with ATZ were calculated to estimate the potential value of treatment with ATZ compared to existing BSC.

### 2.2 Inputs

Model inputs were sourced from either clinical trial data, published studies identified through a targeted literature review, or other publicly available sources. When multiple suitable datasets were identified, input data were selected based on recency, size, and generalizability of the dataset to the US population. Model inputs were included as means with variance parameters (95% confidence interval [CI] or standard deviation [*SD*]); in the absence of reported variance, ±10% was assumed. Base case epidemiological, clinical, and cost inputs, along with their variance, distributions, and sources are presented in Table 1.

**TABLE 1:** Model inputs, distributions, and data sources for the base case

Input		Mean input	95% CI	Distribution	Source
Epidemiology inputs					
Overall US Population		332,601,000	NA	NA	US Census 2020^14^
Proportion of US population aged ≥20		76.26%	68.63%, 83.89%	Beta	
eNSCLC incidence rate aged ≥20 years		0.01309%	0.01298%, 0.1319%	Normal	SEER Stat Incidence Session (2000–2018)^27^
Stages II‐IIIA		87.76%	84.47%, 89.79%	Beta	Felip et al.^11^
Proportion of patients with resectable tumors		80.60%	72.54%, 88.66%	Beta	Genentech Data on File^17^
Proportion of patients receiving adjuvant chemotherapy		44.70%	40.23%, 49.17%	Beta	MacLean et al.^9^
Proportion of patients tested for PD‐L1		77.00%	69.30%, 84.70%	Beta	Velcheti et al.^28^
PD‐L1 Status		53.23%	50.15%, 56.32%	Beta	IMpower010 Trial^17^
Clinical inputs					
*Recurrence rates, BSC*					
Year 1		25.29%	19.51%, 31.07%	Normal	Felip et al.^11^
Year 2		39.02%	32.48%, 45.55%		
Year 3		51.78%	59.27%, 44.29%		
Year 4		53.73%	47.55%, 56.65%		Felip et al.^11^; Cai et al.^2^
Year 5		55.10%	50.10%, 60.20%		Cai et al.^2^
*Mortality rate, BSC*					
Year 1		5.05%	2.14%, 7.96%	Normal	IMpower010 Trial^17^
Year 2		13.02%	8.52%, 17.51%		
Year 3		21.47%	15.67%, 27.27%		
Year 4		27.37%	20.03%, 34.70%		
Year 5		38.80%	30.70%, 42.90%		Cai et al.^2^
*ATZ HR, base case*					
Overall survival		0.77	0.51, 1.17	Log‐normal	Felip et al. 2021^11^
Disease‐free survival		0.66	0.50, 0.88	Log‐normal	
*Recurrence type*					
% locoregional only	ATZ	49.32%	37.85%, 60.78%	Beta	IMpower010 Trial^17^
	BSC	44.12%	34.48%, 53.75%		
% distant only	ATZ	38.36%	27.20%, 49.51%		
	BSC	39.22%	29.74%, 48.69%		
% locoregional and distant	ATZ	12.33%	6.00%, 19.87%		
	BSC	16.67%	9.43%, 23.90%		
% CNS only	ATZ	10.96%	3.79%, 18.12%		
	BSC	11.76	2.21%, 18.02%		
Cost inputs					
*Direct annualized medical costs*					
Office visits	localized	$34,649	$31,245, $38,052	Log‐normal	Gildea et al.^16^
	metastatic	$62,455	$61,897, $62,922		
Hospital outpatient visits	localized	$43,520	$39,802, $47,238		
	metastatic	$78,947	$78,166, $79,166		
ER visits	localized	$608	$556, $660		
	metastatic	$1510	$1459, $1562		
Inpatient visits	localized	$61,730	$39,802, $47,238		
	metastatic	$122,223	$119,713, $124,733		
Other	localized	$4080	$3731, $4428		
	metastatic	$8727	$8410, $9045		
*Direct post‐recurrence drug costs*					
Proportion using CIT post‐recurrence	ATZ	11.0%	3.8%, 18.1%	Beta	IMpower010 Trial^17^
	BSC	35.3%	26.0%, 44.6%		
Proportion using chemotherapy post‐recurrence	ATZ	54.8%	43.4%, 66.2%		
	BSC	46.1%	36.4%, 55.8%		
CIT cost		$165,603	$149,042, $182,163	Log‐normal	Genentech Data on File^17^
Chemotherapy cost		$4173	$3755, $4590		
*Indirect costs*					
Proportion working		62.23%	56.00%, 68.45%	Beta	US Bureau of Labor Statistics^29^
Proportion taking short term leave		38.00%	34.20%, 41.80%		Andreas et al.^30^
Proportion taking long term leave		10.00%	26.00%, 44.60%		Andreas et al.^30^
Proportion of male patients		66.90%	60.21%, 73.59%		Felip et al.^11^
Male daily wage		$219.00	$197.10, $240.90	Log‐normal	US Bureau of Labor Statistics^18^
Female daily wage		$178.00	$160.20, $195.80		US Bureau of Labor Statistics^18^
*Terminal care costs*		$86,904	$84,221, $89,594	Log‐normal	Sheehan et al. ^19^

Abbreviations: ATZ, atezolizumab; BSC, best supportive care; CI, confidence interval; CIT, cancer immunotherapy; CNS, central nervous system; eNSCLC, early non‐small cell lung cancer; ER, emergency room; HR, hazard ratio; NA, not applicable; PD‐L1, programmed death ligand 1. SEER, Surveillance Epidemiology and End Results.

#### 2.2.1 Epidemiological inputs

The number of patients included in the model was calculated through an epidemiological cascade in which the overall US population (328 million)^14^ was filtered to align with the ATZ FDA label for patients with eNSCLC.^12^ These inputs included patients' age, age‐specific eNSCLC incidence, NSCLC stage, proportion of patients with resectable tumors receiving adjuvant chemotherapy, and PD‐L1 status (Table 1).

#### 2.2.2 Clinical inputs

Clinical inputs were informed by the IMpower010 trial, which had a median follow‐up of 32.2 months,^11^ and data from a retrospective observational study that included patients with resected stage II‐IIIB NSCLC who received adjuvant therapy between 2008 and 2017 at US Oncology Network clinics.^2^ Recurrence rates for BSC for years 1, 2, and 3 were estimated based on the DFS Kaplan–Meier (KM) curve from the IMpower010 trial after excluding DFS events that were associated with death as the first event.^11^ Year 4 recurrence rate was calculated as the average between the rate observed in the IMpower010 trial and that reported by the real‐world study.^2^ The recurrence rate for year 5 was taken from the same real‐world study.^2^ This approach was taken to ensure a continuously increasing recurrence rate (i.e., monotonicity) across years 1–5. Recurrences were classified as locoregional, distant, or both locoregional and distant using the proportions reported in IMpower010.^15^

Similarly, mortality rates for years 1, 2, 3, and 4 were estimated based on the BSC OS KM curve from the IMpower010 trial.^11^ As there were no issues with monotonicity for mortality rates, the mortality rate for year 5 was taken directly from the real‐world study.^2^ To calculate the rate of recurrence and mortality for ATZ, HRs from the IMpower010 trial were then applied to the BSC KM curve; recurrence and mortality rates for ATZ were assumed to be constant over the 5‐year period.

#### 2.2.3 Cost inputs

The model considered direct costs, indirect costs, and terminal care costs. Direct costs included annualized medical costs (i.e., office visits, outpatient visits, emergency room [ER] visits, inpatient visits), and one‐time post‐recurrence drug treatment costs. Annualized medical costs were sourced from a retrospective US real‐world study using pharmacy and medical claims linked to clinical cancer registry data in a large NSCLC commercial population.^16^ Locoregional recurrence costs were assumed to be similar to average stage I‐III NSCLC costs, whereas distant, locoregional + distant, and CNS recurrence costs were assumed to be similar to stage IV NSCLC costs (Table 1). Post‐recurrence drug costs for ATZ and BSC were determined based on the proportion of patients in the IMPower010 trial receiving cancer immunotherapy (CIT) or chemotherapy following recurrence.^15^ These costs were calculated as the average cost for first‐line metastatic NSCLC treatments based on US treatment guidelines and weighted by internal market share estimates (Table S1).^17^ The first‐line treatment cost included the cost of the drug itself based on wholesale acquisition cost (WAC) and average treatment duration observed in clinical trials, that is, number of cycles until progression or unacceptable toxicity, administration costs, and costs associated with adverse events. Indirect costs from productivity loss, including short‐term and long‐term leave, were assumed to be the same regardless of recurrence type and were calculated based on average gender‐specific US daily wages (Table 1).^18^ Terminal care costs, accrued in the 6 months prior to death, were sourced from an analysis of stage I‐IV lung cancer patients (median age of 75).^19^ All costs were inflated to 2021 US dollar (USD), using the Consumer Price Index inflation calculator.^20^

#### 2.2.4 Model outputs

Key model outcomes included reductions in clinical events and costs, reported as mean outcomes with 95% CIs. Clinical outcomes included the number of recurrences by type (locoregional, distant, or locoregional+distant, and CNS), number of deaths, and life years saved. Cost outcomes included direct medical costs (office visits, outpatient, ER Visits, inpatient, other, and pharmacy), indirect costs (work productivity loss), and terminal care costs. Model outcomes were reported by year and as cumulative costs over 5 years.

#### 2.2.5 Calculations

A total of 5000 simulations were used to estimate model outcomes. With each simulation, input values were selected from either beta (for proportions), a normal (for population‐level variables) or log‐normal (for HRs and costs) distributions (Table 1).

#### 2.2.6 Scenario analyses

Two scenario analyses were explored (Table S2). The first scenario explored direct costs reflective of a subset of patients aged ≥65 years (i.e., a Medicare population), where the incremental cost of recurrence (in terms of outpatient visits, inpatient visits and other costs) was taken from a real‐world study of eNSCLC among Medicare enrollees (Table S3).^21^ The second scenario explored an increase in the proportion of patients receiving adjuvant chemotherapy post‐resection, as more patients may be willing to receive chemotherapy to be eligible for newer immune‐oncology therapies that significantly improve DFS. An increase of 10% from the base in the proportion of patients receiving adjuvant chemotherapy post‐rejection was explored (Table S2).

## 3 RESULTS

**FIGURE 1:**
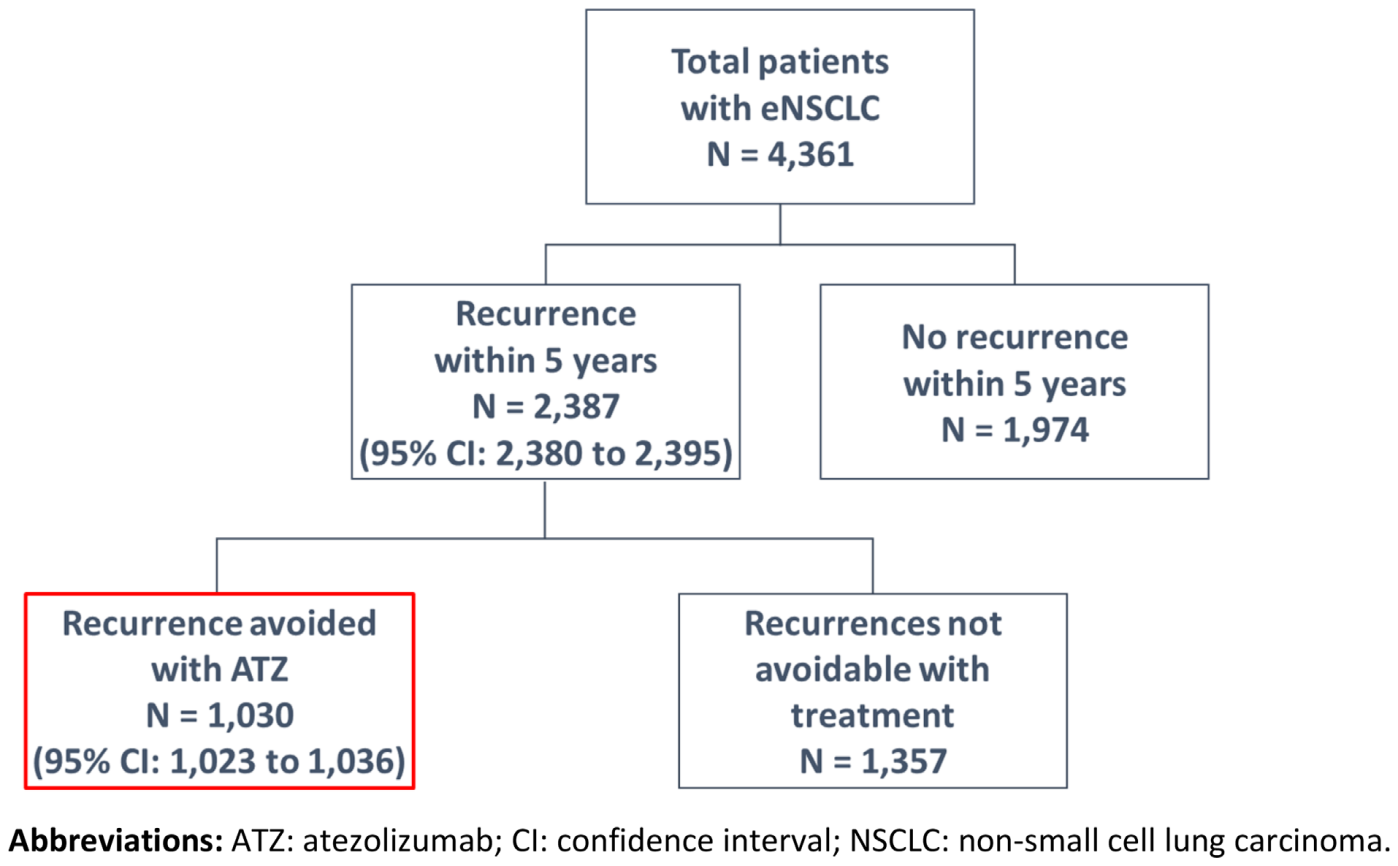
Estimated number of patients with eNSCLC and recurrences avoided with ATZ over 5 years.

**FIGURE 2:**
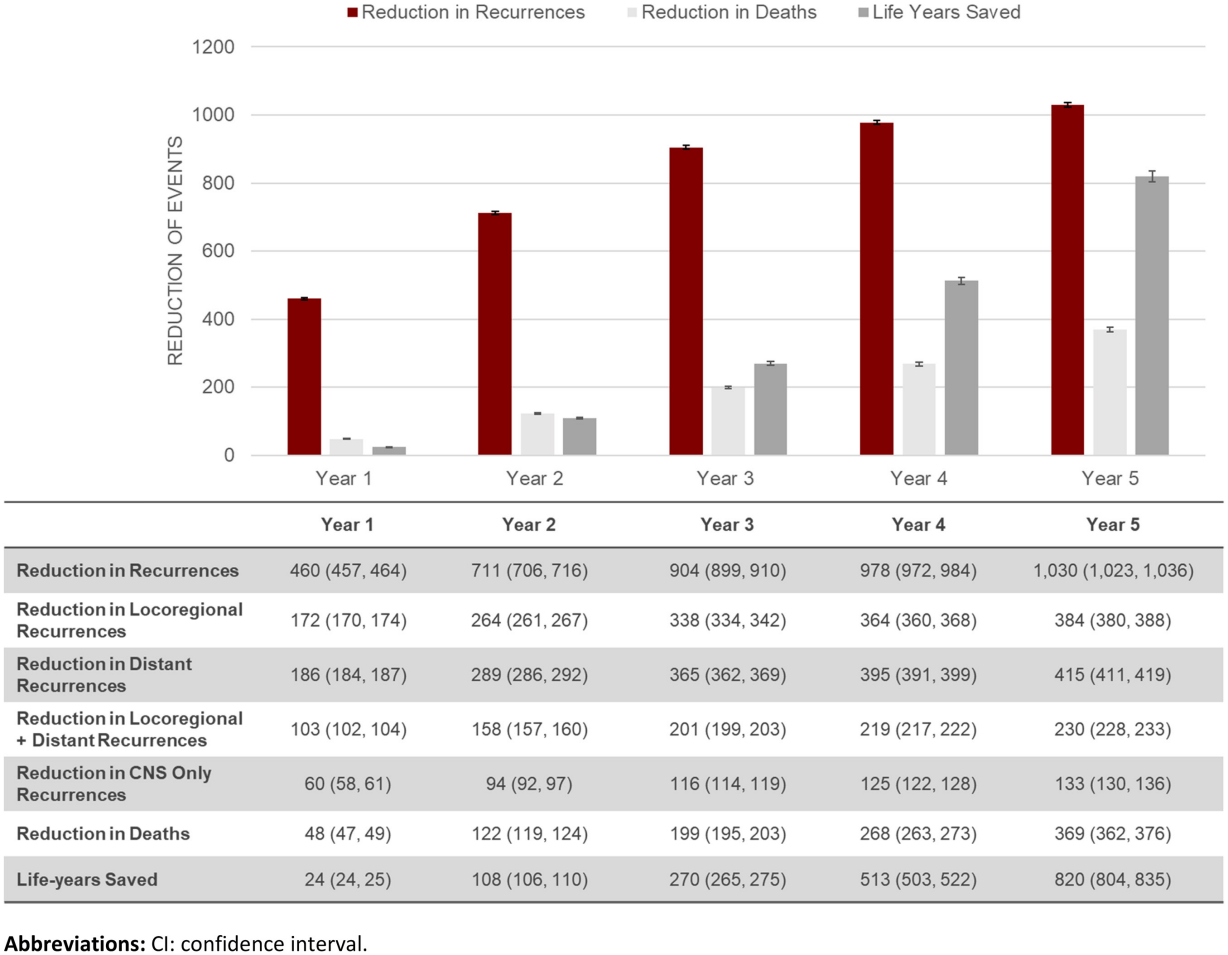
Estimated cumulative clinical outcomes, mean (95% CI).

An estimated 4361 adults with resected stage II to IIIA eNSCLC with PD‐L1 expression of ≥1% were expected to initiate adjuvant chemotherapy in the United States in any given year. Of these, an estimated 2387 (95% CI: 2380, 2395) would have a recurrence within 5 years if receiving BSC. Following the introduction of ATZ, treatment with ATZ would result in 1030 (95% CI: 1023, 1036) recurrences and 369 (95% CI: 362, 376) deaths avoided, over a 5‐year time horizon (Figures 1 and 2). Among the 1030 recurrences avoided, 415 (95% CI: 411, 429) would be distant recurrences including 133 (95% CI: 130, 136) CNS only recurrences; 230 (95% CI: 228, 233) would be locoregional and distant recurrences. The avoidance of recurrences and deaths would result in an estimated 820 (95% CI: 804, 835) life years saved (Figure 2).

**FIGURE 3:**
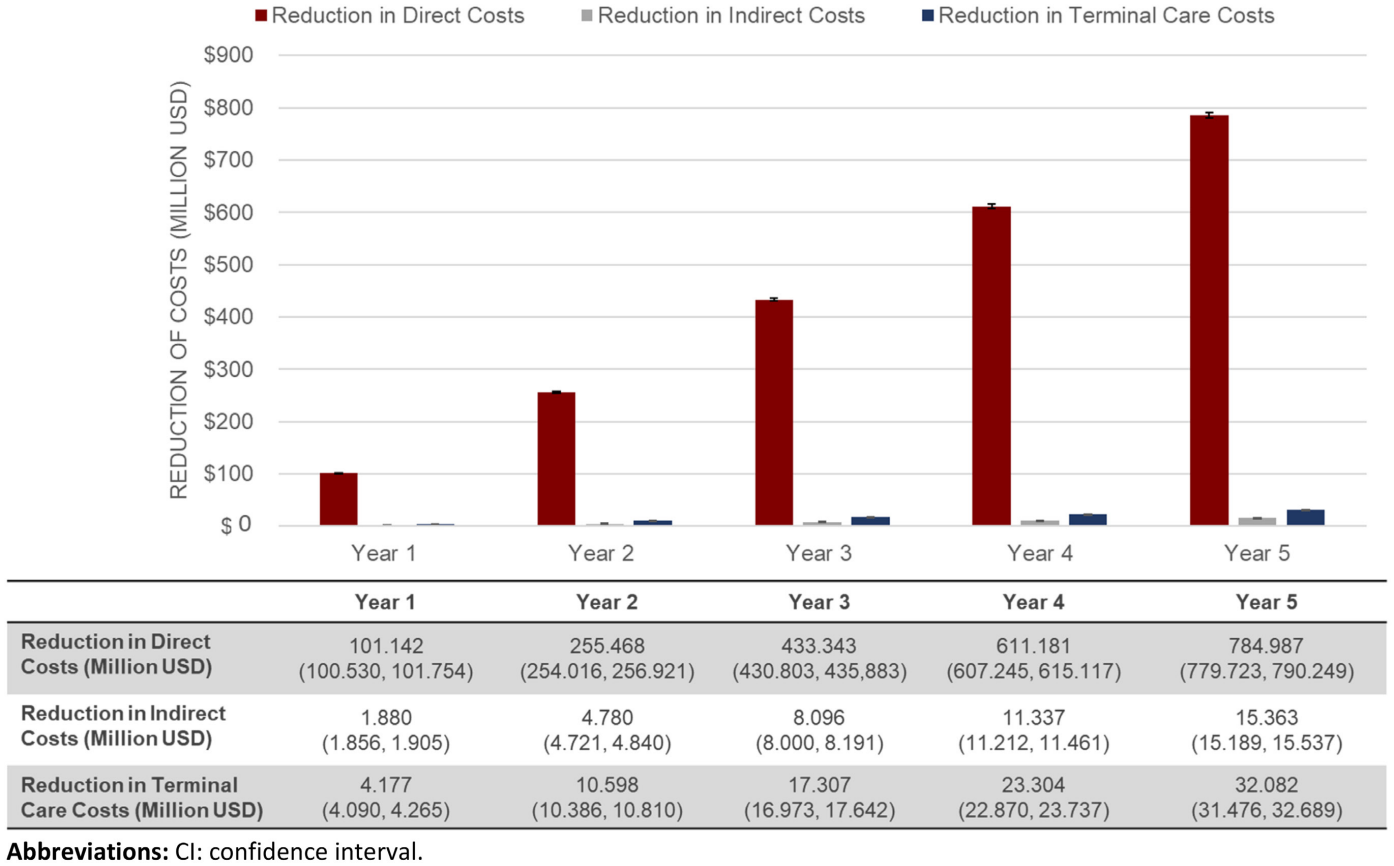
Estimated cumulative cost outcomes, mean (95% CI).

**FIGURE 4:**
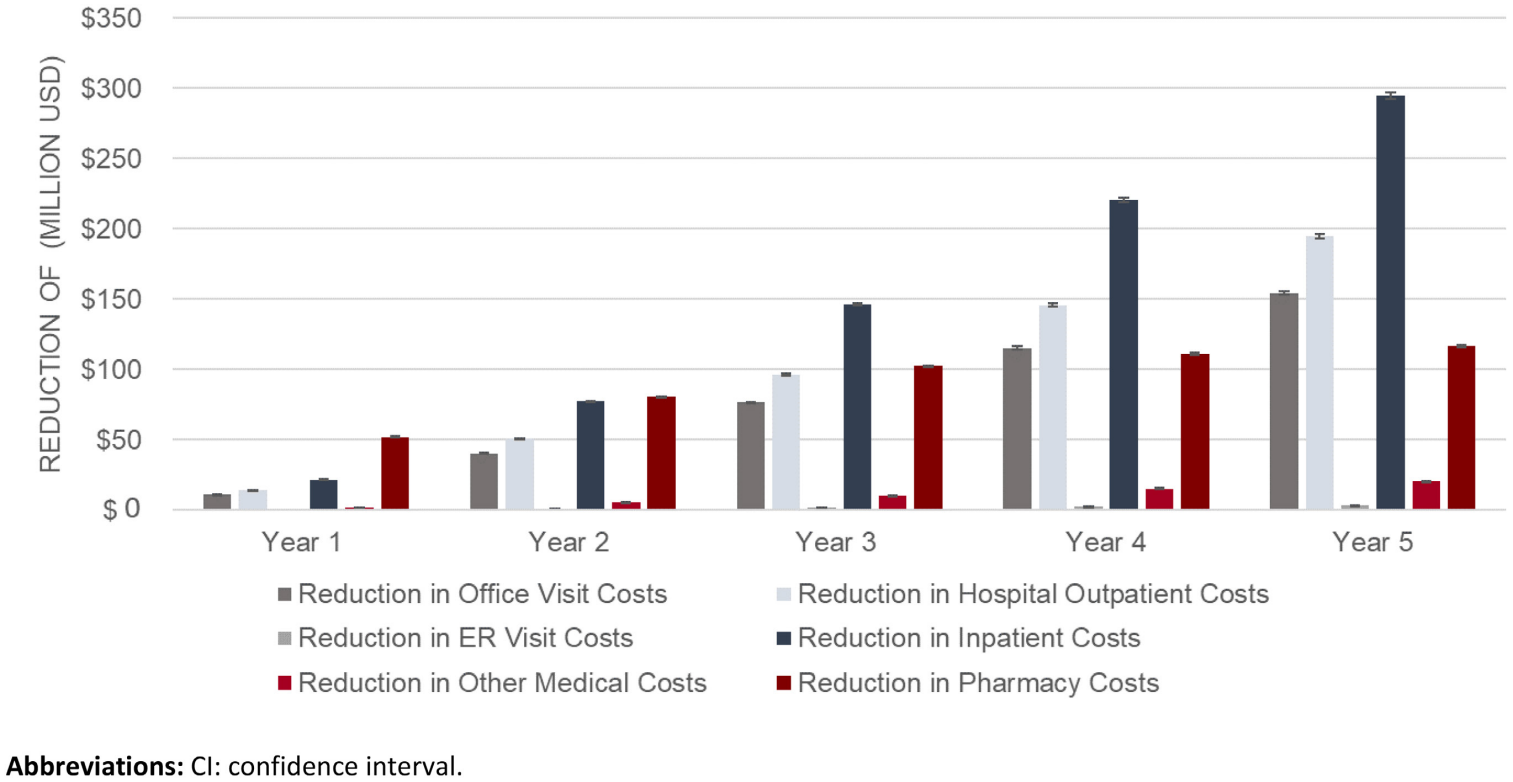
Estimated cumulative direct medical cost outcomes, mean (95% CI).

Treatment with ATZ would result in an estimated cumulative recurrence‐related cost reduction of $785 million (95% CI: $779 million, $790 million) in direct medical costs over 5 years (Figure 3; Table S4). Within the first year following the introduction of ATZ alone, a substantial reduction in recurrence‐related costs of over $100 million was estimated (Figure 3). Over the 5‐year time horizon, the greatest savings were observed in inpatient ($295 million) and hospital outpatient (195 million) costs (Figure 4; Table S4). Further savings were observed in indirect ($15 million) and terminal care ($32 million) costs (Figure 3).

### 3.1 Scenario analyses

When considering only a Medicare patient population (≥65 years), the model estimated a total of 3815 patients eligible for adjuvant ATZ treatment in any given year. Over 5 years, treatment with ATZ would avoid 903 (95% CI 898, 909) recurrences and 323 (95% CI 317, 330) deaths in a Medicare patient population. When using Medicare reimbursement rates (which are lower than those of commercial plans), the model estimated recurrence‐related cost savings of $371 million, $13 million, and $28 million in direct, indirect, and terminal care costs, respectively (Table 2).

**TABLE 2:** Clinical and cost outcomes of scenario analyses (mean and 95% CI)

	Clinical outcomes^a^		Cost outcomes^a^		
Scenario	Recurrences	Deaths	Direct costs	Indirect costs	Terminal care costs
Base Case	1027 (1020, 1033)	373 (366, 380)	$781,278,450 ($775,882,245, $786,674,656)	$15,461,269 ($15,289,248, $15,633,290)	$32,419,567 ($31,816,556, $33,022,577)
Medicare population only	903 (898, 909)	323 (317, 330)	$370,652,369 ($368,308,289, $372,996,449)	$13,471,673 ($13,319,900, $13,623,448)	$28,072,104 ($27,543,781, $28,600,429)
Increase adjuvant chemotherapy by 10%	1138 (1132, 1145)	407 (400, 415)	$866,429,591 ($860,497,302, $872,361,881)	$16,975,379 ($16,782,530, $17,168,227)	$35,397,841 ($34,724,681, $36,071,000)

^a^ Figures presented are reductions in clinical events and costs.

When the proportion of patients receiving adjuvant chemotherapy was increased by 10% from the base case, the number of those eligible for treatment with ATZ also increased. In this scenario, the estimated number of patients initiating treatment with ATZ in any given year was 4797. Compared with base case, greater reductions in both the number of recurrences and deaths with 1138 (95% CI 1132, 1145) and 407 (95% CI 400, 415), respectively, as well as in recurrence‐related direct, indirect, and terminal care costs ($866 million, $17 million, and $35 million, respectively) were estimated (Table 2).

## 4 DISCUSSION

The clinical and economic impact of recurrences among eNSCLC patients in the United States is significant. In this study, we found that initiating an estimated 4361 eligible eNSCLC patients on adjuvant ATZ per year would avoid 1030 recurrences and 369 deaths (vs. BSC) over a cumulative 5‐year period. The avoidance of these recurrences would result in a reduction of over $800 million in recurrence‐related costs, primarily due to reduced direct costs. Cost savings would also be observed among a Medicare‐only patient population, with total reductions of over $384 million. These results highlight the reduction in both the clinical and economic burden among eNSCLC patients in the United States after FDA approval of ATZ.

Recurrences are common among patients with eNSCLC treated with adjuvant platinum‐based chemotherapy post‐resection, and are associated with substantial health care resource utilization.^2^ A recent US‐based retrospective study found that patients in the community oncology setting with stage II to IIIB NSCLC experiencing a recurrence had significantly increased health care resource use requiring more hospitalizations and ER visits (*p* < 0.0001 for both) after the recurrence than before.^2^ This study quantifies in dollar terms the avoidance in health care spent due to the reductions in health care resource utilization associated with recurrences among eNSCLC patients. An older study on patients with lung cancer reported that patients who experience a recurrence and require multiple lines of treatment incurred 2.5‐fold higher overall costs than patients only receiving the initial treatment regimen.^22^ Similar observations have been made for patients with recurrent breast cancer who incurred an over sixfold increase in costs in the 12‐month post‐recurrence period compared with the 12‐month pre‐recurrence period.^23^ Therefore, novel treatments that reduce the risk of recurrence are expected to significantly reduce the total cancer‐associated costs. The precise economic impact of cancer recurrences is difficult to quantify as data on cancer recurrences are not systematically collected in the United States.^24^ However, a 2019 study analyzing health care expenditure of cancer care in the United States identified lung cancer as the most costly type of cancer,^25^ suggesting that any cost reduction in the associated costs will lead to significant savings to the health care budget.

This model did not consider the cost of ATZ treatment or administration nor the cost of managing ATZ‐associated adverse events. Thus, the clinical and economic benefit observed is the impact associated with the prevention of recurrences due to ATZ treatment rather than a reduction in the total cost of care. Nevertheless, this analysis demonstrates that any adoption of ATZ in eligible patients is associated with significant reduction in costs through recurrences avoided. Further, cost estimates are likely underestimated due to the exclusion of caregiver costs and potential increases in the number of eligible patients in the future, either due to potential improvements in lung cancer screening rates or adjuvant chemotherapy utilization given the advent of more effective treatment for eNSCLC patients. It should also be noted that a recent study which did consider treatment costs in its cost‐effectiveness model found ATZ to be cost‐effective versus BSC for the adjuvant treatment of patients with resected PD‐L1+ stage II‐IIIA NSCLC, further supporting utilization of this regimen as the new standard of care in this setting.^26^

As with all economic models, this study has limitations. The cost of a recurrence in eNSCLC is not well‐established in the literature and model cost inputs were compiled from various sources. In addition, rates of recurrence in the real‐world may differ from those observed in clinical trials. Moreover, as the estimated number of reduction of deaths and potential life‐years saved is based on overall survival data from IMpower010, which were not yet mature. However, the Monte Carlo simulation accounted for this uncertainty by using mean and CI values and sampling probabilistically from input value distributions over the 5000 simulations. Lastly, given the recent approval of ATZ in the adjuvant setting, future research is needed using real‐world databases with long‐term follow‐up data available to validate the long‐term outcomes in this study.

This study demonstrates that ATZ represents a valuable new therapeutic option for the adjuvant treatment of resected eNSCLC patients and can aid various stakeholders facing resource constraints to make appropriate treatment‐related decisions for eNSCLC patients. Treatment with ATZ may prevent a significant number of recurrence events and deaths, thus reducing the economic burden in this patient population.

## AUTHOR CONTRIBUTIONS

**Rishika Sharma:** Conceptualization (equal); data curation (equal); formal analysis (equal); methodology (equal); writing – original draft (equal); writing – review and editing (equal). **Sarika Ogale:** Conceptualization (equal); formal analysis (equal); methodology (equal); project administration (equal). **Nathaniel J. Smith:** Conceptualization (equal); data curation (equal); formal analysis (equal); methodology (equal); writing – original draft (equal). **Janet Lee:** Conceptualization (equal); formal analysis (equal); methodology (equal); project administration (equal); writing – original draft (equal); writing – review and editing (equal).

## FUNDING INFORMATION

This work was funded by Genentech Inc.

## CONFLICT OF INTEREST

JSL and SO were full‐time employees of Genentech Inc. during the conduct of this study. RS and NJS have no conflicts to declare.

## Supporting information


Tables S1‐S4.

## Data Availability

N/A.
