# Supplementary material for: Estimating recurrences prevented and costs avoided with atezolizumab in early non‐small cell lung cancer in the United States
Source: Cancer Med. 2022 Nov 24;12(6):7450–8. doi: 10.1002/cam4.5462 (PMC10067035; doi:10.1002/cam4.5462)
Supplement: Supplementary file 1 — Tables S1‐S4. [file CAM4-12-7450-s001.docx]

# SUPPLEMENTARY INFORMATION

**Table S1: Weighted average treatment cost calculation for 1L metastatic treatment with CIT or chemotherapy^17^**

| **Scenario** | **Number of patients^a^** | **Total treatment cost^b^** | **Weighted average cost^c^** |
| --- | --- | --- | --- |
| **CIT** | | | |
| Atezolizumab | 4 | $473,383 | - |
| Pembrolizumab | 37 | $3,766,314 | - |
| Atezolizumab + carboplatin + albumin-bound paclitaxel | 3 | $280,799 | - |
| Atezolizumab + bevacizumab + carboplatin + paclitaxel | 3 | $732,559 | - |
| Pembrolizumab + paclitaxel + carboplatin | 29 | $2,820,959 | - |
| Pembrolizumab + pemetrexed + carboplatin | 78 | $17,428,881 | - |
| Total | 154 | $25,502,895 | $165,603 |
| **Chemotherapy** | | | |
| Pemetrexed + carboplatin | 1 | $41,338 | - |
| Paclitaxel + carboplatin | 54 | $188,164 | - |
| Total | 55 | $229,509 | $4,173 |

**Abbreviations:** 1L, first line; CIT, cancer immunotherapy.

^a^Based on internal market share estimates for 1L metastatic NSCLC treatment.

^b^Includes drugs cost based on Wholesale Acquisition Costs (Medispan PriceRx, last accessed April 2020) and average treatment duration, drug administration costs, and adverse event management costs.

^c^Calculated by dividing the total treatment cost by the total number of patients.

**Table S2: Scenario Analyses**

| **Scenario** | **Description** |
| --- | --- |
| Medicare population | Model only considering patients age 65+ rather than 20+ (base case) with an annualized cost of recurrence of $84,445 for localized and metastatic recurrences based on a real-world study conducted by GENENTECH rather than $144,586 for localized and $273,863 for metastatic recurrences (base case). Cost inputs for office and ER visits were identical to the base case. |
| Increased adjuvant chemotherapy proportion (+10%) | Proportion of patients receiving adjuvant chemotherapy set to 49.17% instead of 44.70% (base case) |

**Abbreviations:** ER, emergency room.

**Table S3: Inputs for the Medicare Scenario**

| Input | | Mean Input | 95% CI | Distribution | Source |
| --- | --- | --- | --- | --- | --- |
| *Epidemiology inputs* | | | | | |
| Proportion of US population aged 60-69 | | 11.74% | 10.57%, 12.91% | Beta | US Census 2020^14^ |
| Proportion of US population aged 70-79 | | 7.29% | 6.65%, 8.02% |  |  |
| Proportion of US population aged 80-84 | | 1.85% | 1.67%, 2.04% |  |  |
| Proportion of US population aged 85+ | | 1.82% | 1.64%, 2.00% |  |  |
| eNSCLC incidence rate aged 60-69 years | | 0.03069% | 0.03008%, 0.03131% | Normal | SEER Stat Incidence Session (2000-2018)^27^ |
| eNSCLC incidence rate aged 70-79 years | | 0.06784% | 0.06661%, 0.06908% |  |  |
| eNSCLC incidence rate aged 80-84 years | | 0.07332% | 0.07166%, 0.07499% |  |  |
| eNSCLC incidence rate aged 85+ years | | 0.04226% | 0.041085%, 0.043468% |  |  |
| *Annualized medical costs* | | | | | |
| Office visits | localized | $34,649 | $31,245, $38,052 | Log-normal | Gildea^16^ |
|  | metastatic | $62,455 | $61,897, $62,922 |  |  |
| Hospital outpatient visits | localized | $12,168 | $10,951, $13,385 |  | Lee^21^ |
|  | metastatic |  |  |  |  |
| ER visits | localized | $608 | $556, $660 |  | Gildea^16^ |
|  | metastatic | $1,510 | $1,459, $1,562 |  |  |
| Inpatient visits | localized | $26,172 | $23,555, $28,789 |  | Lee^21^ |
|  | metastatic |  |  |  |  |
| Other | localized | $10,848 | $9,763, $11,933 |  | Lee^21^ |
|  | metastatic |  |  |  |  |

**Abbreviations:** CI, confidence interval; ER, emergency room.

**Table S4: Cost Outcomes by Category, Base Case**

| **Costs (USD)** | **Mean Outcome** | **95% CI** |
| --- | --- | --- |
| **Direct Costs** |  |  |
| Total Direct Cost Reduction | $784,986,982 | $779,724,769; $790,249,195 |
| Office Visit Cost Reduction | $154,454,417 | $153,302,973; $155,605,861 |
| Hospital Outpatient Cost Reduction | $194,851,327 | $193,398,791; $196,303,863 |
| ER Visit Cost Reduction | $3,461,931 | $3,436,223; $3,487,638 |
| Inpatient Visit Cost Reduction | $294,950,509 | $292,756,657; $297,144,360 |
| Other Medical Cost Reduction | $20,675,059 | $20,521,423; $20,828,694 |
| Post-recurrence Drug Costs Reduction | $116,593,737 | $115,887,048; $117,300,426 |
| **Indirect** **Costs** |  |  |
| Total Indirect Cost Reduction | $15,363,049 | $15,189,227; $15,536,872 |
| **Terminal Care Costs** |  |  |
| Total Terminal Cost Reduction | $32,082,493 | $31,475,621; $32,689,364 |

**Abbreviations:** CI, confidence interval; ER, emergency room.
